# How the Human–Artificial Intelligence (AI) Collaboration Affects Cyberloafing: An AI Identity Perspective

**DOI:** 10.3390/bs15070859

**Published:** 2025-06-25

**Authors:** Jin-Qian Xu, Tung-Ju Wu, Wen-Yan Duan, Xuan-Xuan Cui

**Affiliations:** 1 Business School, Harbin Institute of Technology, Harbin 150001, China; 23sm46181@stu.hit.edu.cn; 2 School of Management, Harbin Institute of Technology, Harbin 150001, China; tjwu@hit.edu.cn (T.-J.W.); 22s010099@stu.hit.edu.cn (X.-X.C.)

**Keywords:** human–AI collaboration, dependence, emotional energy, relatedness, cyberloafing, openness

## Abstract

Collaboration with artificial intelligence (AI) not only improves employees’ work efficiency but also provides them with opportunities to participate in other behaviors. Among the various behaviors that have garnered the attention of organizations, cyberloafing has historically been a focus. Drawing from social identity theory (SIT), this research examines how human–AI collaboration diminishes cyberloafing by fostering AI identity (dependence, emotional energy, relatedness). A three-wave study (N = 381) revealed that AI collaboration strengthened employees’ AI identity, enabling them to recognize their identity as AI collaborators and focus on in-role tasks, thereby reducing cyberloafing. Moreover, the research suggested that openness served as a moderating factor, further amplifying the positive relationship between human–AI collaboration and AI identity. Specifically, employees who exhibit higher levels of openness are more likely to demonstrate heightened AI identity and reduced cyberloafing. Conversely, employees with low openness exhibit less AI identity and more cyberloafing. This research employs SIT in the context of AI collaboration, thereby providing a theoretical foundation and practical guidance for reducing employee cyberloafing in the workplace and promoting organizational development.

## 1. Introduction

Since the advent of ChatGPT, an increasing number of employees in the workplace have begun working with artificial intelligence (AI) (Duan et al., 2024; Wu et al., 2024b). Human–AI collaboration refers to the interaction and cooperation between individuals and AI to efficiently complete work tasks (Kong et al., 2023). AI collaboration exerts a substantial influence on employees’ behavior and performance (Budhwar et al., 2022; Ma et al., 2024). On the one hand, AI can foster innovative behavior and enhance service performance (Duan et al., 2024; Yan & Teng, 2025). On the other hand, AI collaboration leads to employees’ work withdrawal and counterproductive work behavior (Bai & Zhang, 2025; Meng et al., 2025). Some scholars have expressed concerns that employees use the convenience of AI to perfunctorily perform their work, thereby reducing their efforts and even resulting in laziness during working hours (Saluja et al., 2024). Cyberloafing is a lazy behavior of employees engaging in non-work-related activities during their working time (Gupta et al., 2025; Saluja et al., 2024). It refers to the practice of employees leveraging the internet to address personal matters during working hours, such as sending and receiving private messages or browsing shopping websites (Askew et al., 2014; Hessari et al., 2025; Zahmat Doost & Zhang, 2024). Previous research indicates that cyberloafing reduces individual work engagement and organizational productivity (Tsai, 2023; Hessari et al., 2025).

However, it should be noted that human–AI collaboration emphasizes the complementary advantages between humans and AI (Kong et al., 2023; Jia et al., 2023). It distinguishes AI collaborators from traditional workers, and AI collaborators can achieve higher work efficiency and quality with the help of AI, thereby achieving performance superior to that of traditional workers (Przegalinska et al., 2025; Seeber et al., 2020). This contributes to an enhancement in their identity recognition as members of the AI collaboration group. AI identity refers to employees viewing AI collaboration as an integral component of their identity (Cao et al., 2023; Mirbabaie et al., 2022). Based on social identity theory (SIT), individuals’ conceptual division of their social group identity will affect their subsequent behavior (Guo et al., 2021; Rubin et al., 2023). Because of AI identity, employees may reinvest the resources saved by AI collaboration into work, such as engaging in innovative behavior or learning new skills to improve their abilities (Chowdhury et al., 2022; Duan et al., 2024; Li et al., 2024). This helps employees better complete organizational tasks with AI collaboration, rather than encroaching on work events to engage in cyberloafing.

Specifically, human–AI collaboration can reduce cyberloafing by influencing the three dimensions of AI identity (dependence, emotional energy, and relatedness). Dependence is the perception of the importance of AI (Carter et al., 2020; Mirbabaie et al., 2022). The assistance rendered by AI may lead to an increase in employees’ dependence on it, enabling them to complete more tasks by AI collaboration instead of being distracted by other aspects (Bezrukova et al., 2023; Tussyadiah, 2020). Emotional energy refers to the positive emotions experienced by individuals when collaborating with AI (Cao et al., 2023; Mirbabaie et al., 2022). AI collaboration can free up employees’ time and energy required for work, improve their work experience, and reduce negative emotions (Haesevoets et al., 2021; Huo et al., 2025). This helps employees to maintain a positive state at work and increase their investment (Budhwar et al., 2022; Duan et al., 2024). Relatedness is the degree to which an individual connects with AI (Mirbabaie et al., 2022). Employees enhance their comprehension of AI and acquire advanced AI collaboration competencies, thereby fostering a more profound connection between individuals and AI (Li et al., 2024). AI collaboration may enhance employees’ sense of responsibility and accomplishment, thereby facilitating a more focused approach to their job responsibilities. Therefore, AI identity may encourage employees to behave in accordance with their identity and inhibit cyberloafing.

In comparison with a conventional work environment, AI collaboration has precipitated substantial changes in work methods (Hessari et al., 2025; Li et al., 2024; McPhail et al., 2024). This presents a considerable challenge to the acceptance and adaptability of employees (Seeber et al., 2020; Hai et al., 2025). In the context of AI collaboration, employees should embrace new technologies with openness and actively learn how to communicate and collaborate effectively with AI, rather than sticking to old work modes and mindsets (Samimi Dehkordi et al., 2024). Openness is defined as the degree to which employees accept and adapt to new technologies (Barford & Smillie, 2016; Cugno et al., 2021). Employees who have high openness are more inclined to experiment with new methodologies (Chakraborty et al., 2023; Slobodskaya & Kornienko, 2021). They proactively leverage the resources and assistance offered by AI to enhance work efficiency, develop their own skills, and progress in their careers (Zhang et al., 2025). This ongoing engagement helps foster a stronger sense of AI identity. Conversely, employees who exhibit low openness may be reluctant to accept the assistance of AI, potentially leading to diminished AI identity. Therefore, compared to other traits, openness is particularly important in this process.

Based on SIT, this research aims to explore the effects of human–AI collaboration on cyberloafing from the perspective of AI identity. A three-wave study was designed (N = 381). The results indicated that human–AI collaboration effectively reduced cyberloafing by enhancing AI identity. In addition, openness played a positive moderating role. This research makes both theoretical and practical contributions. Theoretically, we examined the effect of AI collaboration on cyberloafing from the perspective of AI identity, thereby expanding the scope of SIT. Second, this research offers a clarification of the definition of AI identity and its mediating role. Third, the moderating role of openness in facilitating AI collaboration was underscored. Practically, organizations and managers can effectively mitigate employees’ non-work-related behavior by fostering a stronger sense of AI identity. Managers can enhance employees’ dependence on AI through training. Managers should understand and address employees’ emotional needs, thereby fostering an emotional connection with AI. They can enhance the relatedness between employees and AI through optimized interaction design. Furthermore, organizations and managers should prioritize the cultivation and selection of employees who exhibit openness traits.

## 2. Theoretical Framework and Hypotheses

### 2.1. Social Identity Theory

Social identity theory (SIT), originally developed by Tajfel and Turner (1979), posits that individuals derive their self-concept from membership in social groups, which influences their attitudes and behaviors in organizational contexts (Ashforth & Mael, 1989; Guo et al., 2021; Hornsey, 2008). At its core, SIT suggests that individuals often categorize themselves and others into diverse social groups based on shared characteristics, such as ingroups and outgroups (Brown, 2000; Stets & Burke, 2000). In the workplace, this theory explains how employees develop professional identities (Rubin et al., 2023; Scheifele et al., 2021). The theory’s three fundamental processes—social categorization, social identity, and social comparison—have been particularly valuable for understanding technology adoption (Guo et al., 2021; Shao et al., 2023).

Social categorization is defined as the process by which individuals organize themselves and others into distinct groups based on shared characteristics or attributes (Guo et al., 2021). This categorization provides a foundation for individuals to comprehend the social environment and their own identity (Adam et al., 2021; Scheifele et al., 2021). Social identity is defined as the sense of self-identity that individuals gain through group belonging. When individuals categorize themselves into a certain group, they internalize the characteristics, values, and behavior patterns of the group as part of their identity (Rubin et al., 2023; Shao et al., 2023). Social comparison refers to the process by which individuals develop self-esteem and self-worth by comparing their group with other groups, which helps individuals enhance their sense of identity as members of the group (Adam et al., 2021; Guo et al., 2021). In this research, we sought to expand the application scope of SIT to the domain of human–AI collaboration.

### 2.2. Human–AI Collaboration and Cyberloafing

Human–AI collaboration refers to the interactive and cooperative process between humans and AI, aimed at efficiently accomplishing work tasks (Kong et al., 2023). AI collaboration has a profound effect on people’s work (Budhwar et al., 2022; Duan et al., 2024). Individuals collaborating with AI no longer work with human partners as in traditional jobs; their teammates or partners have become AI (Seeber et al., 2020). This is a major basis for distinguishing people collaborating with AI from traditional workers. When employees collaborate with AI, AI significantly improves individuals’ work efficiency and quality and also gives them more time to engage in creative work (Duan et al., 2024; Wu et al., 2024b). AI collaboration may foster a favorable perception of the group among employees. As employees derive greater benefit from AI collaboration, they may cultivate a heightened sense of belonging within the AI collaboration team. Additionally, employees who collaborate with AI may perceive their group to exhibit superior performance compared to those who do not collaborate with AI (Vrontis et al., 2022; Zhang et al., 2025). This, in turn, strengthens employees’ social identity, thereby motivating them to adopt behaviors consistent with organizations rather than engaging in counterproductive behaviors (Ashforth & Mael, 1989; Meng et al., 2025; Yan & Teng, 2025).

Cyberloafing, defined as the use of the internet for activities unrelated to work tasks during working hours, is a prevalent issue in the modern workplace, such as browsing social media, watching videos, and shopping (Askew et al., 2014; Blanchard & Henle, 2008). Cyberloafing has been demonstrated to directly diminish the time allocated to work-related tasks, thereby exhausting employees’ limited resources and energy (Chen et al., 2023; Zhang et al., 2020). A significant allocation of time to non-work-related online activities can impede employees’ own task progress and precipitate a decline in productivity (Tandon et al., 2022; Wagner et al., 2012). Consequently, the regulation and control of cyberloafing have consistently been a primary objective for organizations and managers (Koay et al., 2022). According to SIT, employees may perceive themselves as an efficient and innovative group, leading to a strong sense of identity and responsibility (Rubin et al., 2023; Guo et al., 2021; Williams et al., 2009). Consequently, employees may allocate more time and energy to tasks that align with their interests, potentially reducing cyberloafing related to feelings of boredom or dissatisfaction. At the same time, the integration of AI into the workplace enables the expeditious delivery of pertinent information and the progressive generation of customized work outcomes through sustained interaction with employees (Huo et al., 2025; Kong et al., 2023; Ma et al., 2024). These outcomes are more aligned with employees’ job demands, thereby eliminating their tendency to browse and filter irrelevant information online, thus contributing to a reduction in cyberloafing. Moreover, AI collaboration has been demonstrated to alleviate employees’ workloads (Arslan et al., 2022; Budhwar et al., 2022; Vrontis et al., 2022). By enhancing work efficiency and alleviating work pressure, employees can more effectively focus on core tasks, rather than seeking respite from stress through cyberloafing. Therefore, we hypothesized the following:

**Hypothesis** **1.** *Human–AI collaboration is negatively related to cyberloafing.*

### 2.3. The Mediation of Three Dimensions of AI Identity

Information technology (IT) has profoundly transformed the manner in which humans acquire, process, and disseminate information. The advent of IT prompted Carter and Grover (2015) to conceptualize IT identity. This refers to an individual’s belief that the use of information technology is a component of their self-awareness, and a strong IT identity is sufficient to represent a positive self-identity (Carter & Grover, 2015). However, in the era of digitization, merely considering employees’ IT identity is no longer sufficient for AI collaborative work environments. Advancements in technology have led to a gradual replacement of IT with more sophisticated and potent AI (Dunleavy & Margetts, 2023; Mirbabaie et al., 2022; Spring et al., 2022). The collaboration between humans and AI not only reduces resource consumption and enhances efficiency, but also fosters a comprehensive recognition of individual value through the complementary advantages offered by AI. Therefore, in the context of human–AI collaboration, employees may develop a new identity—AI identity; that is, employees regard collaboration with AI as an integral part of their self-concept (Cao et al., 2023; Mirbabaie et al., 2022).

The conceptualization of AI identity is typically delineated across three dimensions (Carter et al., 2020; Mirbabaie et al., 2022). First is the individual’s dependence on AI, which is the level of dependence on AI in one’s professional life (Cao et al., 2023; Reychav et al., 2019). This dependence may manifest in various domains, including operations, decision support, and information processing. Collaboration with AI in work processes has the potential to enhance work efficiency, thereby increasing employees’ dependence on AI to achieve work objectives. The second dimension is emotional energy, which refers to the positive emotional state experienced by individuals when collaborating with AI (Huang, 2019; Reychav et al., 2019). The experience of positive emotions, increased satisfaction, and enhanced work experience can be attributed to AI’s ability (Duan et al., 2024). In the context of AI collaboration, the role of humans is indispensable, and employees may perceive a sense of control and security as a result (Lv et al., 2024). Relatedness is also one of the dimensions of AI identity, defined as the extent to which individuals associate themselves with AI (Carter et al., 2020; Reychav et al., 2019). Employees may perceive a gradual dissolution of the boundaries between themselves and AI, as both parties become equally essential for the completion of work tasks. Concurrently, employees who collaborate with AI regard it as an indispensable aid in their work, in comparison to employees who do not collaborate with AI (Reychav et al., 2019). So we hypothesize the following:

**Hypothesis** **2.** *Human–AI collaboration is positively related to dependence (a), emotional energy (b), and relatedness (c).*

The sense of identification that employees generate when collaborating with AI is crucial for improving their subsequent behavior (Kopp et al., 2021). When employees become dependent on AI, they may prioritize tasks that involve collaboration with AI, potentially leading to a reduction in engagement with non-work-related network activities. This increased dependence may also stem from the belief that AI collaboration can enhance work efficiency, potentially leading to heightened job satisfaction and a reduction in cyberloafing. Emotional energy refers to the positive emotions and emotional states experienced by employees when they collaborate with AI (Carter et al., 2020; Carter & Grover, 2015). When employees receive positive feedback while collaborating with AI, they develop positive emotions such as confidence and trust, and have a stronger sense of control over their work. This, in turn, has the potential to motivate employees to focus on their work tasks, stimulate their enthusiasm and creativity, and encourage them to explore new work methods and solutions. Consequently, this may also reduce cyberloafing. Employees experience a sense of relatedness with AI during collaboration, which may stem from frequent interactions and a deep understanding of AI functionality and capabilities. The enhanced relationship between employees and AI may encourage increased engagement with and loyalty to their work, leading to heightened awareness of their identity and responsibility in AI collaboration. Consequently, employees may prioritize work-related matters over cyberloafing. Therefore, the establishment of a favorable AI identity among employees is likely to engender behaviors that align with that identity, thereby reducing cyberloafing. We hypothesize the following:

**Hypothesis** **3.** *Dependence (a), emotional energy (b), and relatedness (c) mediate the relationship between human–AI collaboration and cyberloafing.*

### 2.4. The Moderating Effect of Openness

Openness plays a pivotal role in the examination of the interplay between AI collaboration and employee cyberloafing. Openness refers to an individual’s acceptance and curiosity toward new things, experiences, and perspectives in terms of thinking, emotions, and behavior (Barford & Smillie, 2016; Tang et al., 2022). Although individuals may exhibit varying degrees of openness at different stages, research has demonstrated that openness affects individuals’ acceptance of and participation in new technologies (Chakraborty et al., 2023; Slobodskaya & Kornienko, 2021). And openness has a significant effect on individual performance (Chakraborty et al., 2023; Mammadov, 2022).

Specifically, openness is a trait that varies among individuals, with those who exhibit high levels of openness being receptive to novel ideas, experiences, and cultural influences (Barford & Smillie, 2016). This proclivity for exploration, experimentation, and acceptance of novel challenges is associated with an increased propensity for creative thinking and behavior (Ayoub et al., 2017; Cugno et al., 2021). Individuals with high levels of openness exhibit proactive coping mechanisms in the face of difficulties and pressures (Williams et al., 2009). These individuals have been found to demonstrate higher psychological resilience, adaptability, and ability to cope with changing social environments and role transitions (Tang et al., 2022). Conversely, individuals with low openness often exhibit a more conservative or resistant stance toward novel concepts and ideas (Cugno et al., 2021).

In the context of AI collaboration, individuals who exhibit high openness demonstrate heightened motivation and capacity for the exploration and utilization of novel technologies. These individuals possess superior cognitive abilities, encompassing memory, attention, language skills, and executive function, among others (Nusbaum & Silvia, 2011). This proclivity for advanced thinking and learning enables them to swiftly acquire the knowledge necessary for effective AI collaboration (Barford & Smillie, 2016; Tang et al., 2022). And employees who exhibit high openness are more likely to accept AI as a collaborative partner, demonstrate higher levels of trust and recognition toward AI, and actively engage with it. They are more inclined to delegate repetitive tasks to AI for processing, enhancing work efficiency (Kong et al., 2023; Ma et al., 2024). Consequently, employees with high openness exhibit a stronger dependence on AI. At the same time, employees with high openness are more likely to experience the positive effects of AI, such as reduced workload and enhanced job satisfaction (Budhwar et al., 2022; Vrontis et al., 2022). They hold a more favorable evaluation and perspective of AI, and can derive emotional energy from AI collaboration. Therefore, employees with a high degree of openness will generate more emotional energy. Furthermore, employees with high openness tend to be curious about new technologies and actively explore the functions of AI, enabling them to develop a more profound understanding of AI and collaborate efficiently (Barford & Smillie, 2016). This makes AI partners indispensable in their professional lives. Thus, openness fosters a closer relatedness between employees and AI. In summary, openness enhances the effect of human–AI collaboration on AI identity. So we hypothesize the following:

**Hypothesis** **4.** *Openness moderates the effects of human–AI collaboration on dependence (a), emotional energy (b), and relatedness (c), such that the relationship is stronger (vs. weaker) when openness is higher (vs. lower).*

From the preceding discussions, we hypothesize that the interaction between human–AI collaboration and openness will enhance employees’ AI identity (Hypothesis 4). AI identity plays a mediating role between human–AI collaboration and cyberloafing (Hypothesis 3). Therefore, it is reasonable to predict that the indirect effects of dependence, emotional energy, and relatedness on cyberloafing will be stronger when employees have a higher level of openness. So we hypothesized the following:

**Hypothesis** **5.** *Openness moderates the indirect effects of human–AI collaboration on cyberloafing through dependence (a), emotional energy (b), and relatedness (c), such that the indirect effects are stronger (vs. weaker) when openness is higher (vs. lower).*

**Figure 1:**
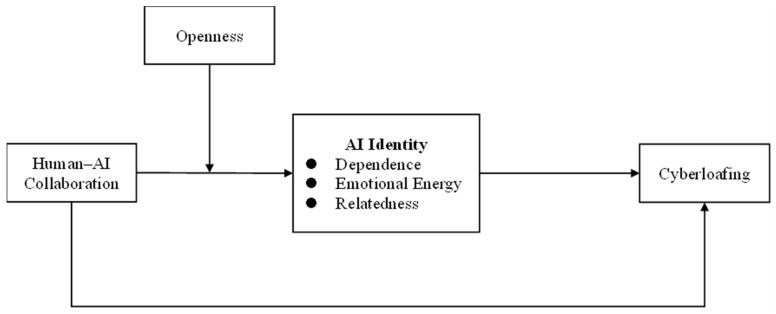
Research model.

In summary, this research proposes that human–AI collaboration reduces cyberloafing by enhancing employees’ AI identity, with openness strengthening this relationship. When employees work with AI that improves efficiency and task quality, they develop a stronger AI identity characterized by three dimensions: dependence (reliance on AI for task completion), emotional energy (positive affect from AI interactions), and relatedness (psychological connection with AI). Dependence on AI decreases time spent on non-work activities by improving task focus; emotional energy enhances individuals’ engagement through positive affective states; and relatedness enhances employees’ closeness to and sense of responsibility for their work through psychological bonding with AI. Therefore, AI identity helps reduce employee cyberloafing. Openness amplifies these effects, as open individuals more readily embrace AI collaboration, develop stronger AI identities, and consequently show a greater reduction in cyberloafing. Therefore, we constructed the model in Figure 1.

## 3. Method

### 3.1. Participants and Design

In contrast to cross-sectional data, multi-wave studies have been shown to reduce common method bias (CMB) (Gupta et al., 2025; Podsakoff et al., 2003). Consequently, a three-wave study was conducted to validate the hypotheses. The participants were recruited through an online platform that is frequently utilized for academic research, Credamo (Wu et al., 2024b). We did not impose any restrictions on the geographical location or organizational type of the participants. The sole prerequisite for participation in this study was AI collaboration. To ensure that participants met the requirements of collaborating with AI, we emphasized the following content at the beginning of the questionnaire: “*The objective of this research is to comprehend the cognitive and behavioral context of employees who collaborate with AI. Individuals not currently engaged in AI collaboration are respectfully requested to refrain from completing the questionnaire.*” At the same time, we assured participants that all survey responses are anonymous and utilized exclusively for academic research purposes, thereby mitigating concerns regarding data disclosure. At time 1 (5 February 2024), 500 questionnaires were disseminated. The participants were asked to provide their demographic information and answer items about human–AI collaboration. Participants who completed the time 1 questionnaire received a reward of RMB 1. Following a two-week interval (time 2; 19 February 2024), a second questionnaire was disseminated to the initial participants, inquiring about their level of AI identity, which encompasses three dimensions (dependence, emotional energy, and relatedness). Participants who completed the second questionnaire received a reward of RMB 1 too. A total of 433 valid questionnaires were collected during time 2, with an effective response rate of 86.6%. At time 3 (time 2; 4 March 2024), participants were requested to report on the cyberloafing they engaged in after collaborating with AI, and they were provided with a compensation of RMB 2. After excluding questionnaires from participants who completed the survey in an unrealistic time frame (<60 s) or an extended time frame (>300 s), the final data set included responses from 381 participants, and the effective response rate was 76.20%. Table 1 presents the correlations and descriptive statistics.

### 3.2. Measures

*Human–AI Collaboration.* We used the Kong et al. (2023) 5-item scale to measure each human’s collaboration with AI in their work. This is a 5-point Likert scale, and one of its items is “*AI participates in my problem-solving process during the work*”. Cronbach’s α was 0.872.

*AI identity.* We measured AI identity with an adapted version of the scale by Carter and Grover (2015). Research by Mirbabaie et al. (2022) confirmed the AI identity scale’s usability. This instrument assesses three dimensions of dependence (e.g., “*Thinking about myself in relation to the AI, I feel needing it*”), emotional energy (e.g., “*Thinking about myself in relation to the AI, I feel energized*”), and relatedness (e.g., “*Thinking about myself in relation to the AI, I feel close to it*”). It ranges from “1 = strongly disagree” to “5 = strongly agree”. The Cronbach’s α values were 0.919 (dependence), 0.888 (emotional energy), and 0.863 (relatedness), respectively.

*Cyberloafing.* Cyberloafing was measured with a 5-point Likert scale developed by Lim and Teo (2005), which has 11 items. These items continuously demonstrate high reliability (Andel et al., 2019). Participants were requested to report how often they engage in various non-work-related online activities during working hours. Sample item is “*At work, I send non-work-related e-mail*”. Cronbach’s α was 0.956.

*Openness.* The 4-item scale of John and Srivastava (1999) was used to evaluate openness. A sample item is “*At work, I have a vivid imagination at work*”. Participants ranked their responses from 1 for “strongly disagree” to 5 for “strongly agree”. Cronbach’s α was 0.884.

*Control variables.* In order to eliminate the possibility of alternative explanations, we controlled several variables that were deemed to potentially impact our dependent variables. These control variables included gender, age, education, and tenure in years (Liang et al., 2024).

At the same time, the Average Variance Extracted (AVE) and Composite Reliability (CR) values of each variable were calculated. It was determined that AVE is greater than the correlation between variables, and CR values are greater than 0.9. This indicates that the scales utilized in this study possess adequate convergent validity and internal consistency.

### 3.3. Data Analysis Process

Confirmatory factor analysis (CFA) is used to verify the consistency between a constructed factor structure and actual data. Amos 24.0 is widely used in CFA. In its graphical interface, we drew the relationship diagram between latent factors and observed variables. Each latent factor is represented by an ellipse, the observed variable is represented by a rectangle, and the factor loading is connected by an arrow to the latent factor and the observed variable. After drawing the relationship diagram, we imported the data and output various statistical indicators, such as IFI, CFI, and GFI values. By observing the values of these indicators, we can judge the goodness of the model fit (Wu et al., 2024a).

Harman’s single-factor test is used to evaluate the effect of common method bias (CMB). This method uses exploratory factor analysis (EFA) to determine whether there is a dominant factor in the data, thereby inferring whether there is CMB (Podsakoff et al., 2003). We used SPSS 25.0 to conduct CFA and moved all research variable data into the variable box of factor analysis. In choosing principal components as the analysis method, there is no need to rotate the factors, and finally, the loading values of all factors can be obtained. By observing the proportion of variance explained by the first factor, we can determine whether the study has serious common method bias problems (Podsakoff et al., 2003).

The analysis of main effects, mediating effects, and moderating effects is a critical method for elucidating the intricate relationship between variables. Mplus 8.3 has emerged as a highly suitable instrument for such analyses, owing to its advanced statistical analysis capabilities and adaptable model construction functionality. For the main-effect analysis, the model included control variables (gender, age, education, and tenure in years), independent variables (human–AI collaboration), and dependent variables (cyberloafing), mainly examining the direct effect between human–AI collaboration and cyberloafing. The mediation-effect analysis added the mediating variable—AI identity (dependence, emotional energy, relatedness)—and formulated a path model of human–AI collaboration→AI identity (dependence, emotional energy, relatedness)→cyberloafing. The moderating-effect analysis necessitated the incorporation of a moderating variable (openness) into the model, along with the construction of an interaction term (human–AI collaboration × openness) with the independent variable (human–AI collaboration). In the analysis of mediation and moderation effects, Mplus 8.3 estimates the confidence interval through the Bootstrap, which generates a large number of samples through 5000 repeated samplings, thereby providing a more robust confidence interval estimate. Therefore, we have verified the hypotheses through the above analysis process, and the specific test results will be elaborated in detail in the subsequent content.

### 3.4. Results

*CFA.* We first checked discriminant validity through CFA by Amos 24.0. The objective of CFA is not to substantiate the veracity of the hypothesis; rather, it is to assess the discriminant validity and the model’s fitness (Wu et al., 2024a). The χ^2^*/df* < 3, RMSEA < 0.08, and other fitting indicators greater than 0.8 indicate that the model has a good fitting effect. So the findings of the CFA demonstrate that the model under consideration in this study exhibits a satisfactory degree of fit (χ^2^*/df* = 2.617, IFI = 0.932, TLI = 0.923, CFI = 0.931, GFI = 0.873, AGFI = 0.846, RMSEA = 0.065). The 40% refers to the critical value of CMB. Furthermore, to avoid the issue of homoscedasticity that may arise from employee self-assessment, the Harman single-factor test was conducted, revealing that the first factor accounted for a mere 25.853% of the variance. This aligns with the stipulated requirement of less than 40% (Podsakoff et al., 2003).

*Main effect.* To test our hypotheses, Mplus 8.3 and 5000 resamples were utilized in the mediation analyses. As shown in Table 2, a considerable negative relationship has been observed between human–AI collaboration and cyberloafing (B = −0.279, *p* < 0.001). Hypothesis 1 was confirmed. This illustrated the potential for human–AI collaboration to facilitate the segmentation of employees, enabling them to reap the benefits of increased productivity and quality, thereby helping them focus on core tasks rather than engaging in cyberloafing (Chen et al., 2023; Duan et al., 2024; Seeber et al., 2020).

*Mediation analyses.* According to Table 2, human–AI collaboration was positively related to dependence (B = 0.228, *p* < 0.01), emotional energy (B = 0.180, *p* < 0.01) and relatedness (B = 0.406, *p* < 0.001). Human–AI collaboration fosters AI identity through three key dimensions (Cao et al., 2023; Carter et al., 2020). Dependence emerges as employees increasingly rely on AI for task completion and decision-making, recognizing its critical role in work processes (Reychav et al., 2019). Emotional energy develops through positive affective experiences during AI collaboration, including satisfaction from enhanced efficiency and problem-solving capabilities (Duan et al., 2024; Huang, 2019). Third, relatedness grows as employees perceive AI as an integral work partner (Mirbabaie et al., 2022; Reychav et al., 2019). AI collaboration fosters an augmentation in employees’ reliance on and association with AI partners, concurrently engendering a positive sentiment. Hypothesis 2a, 2b, and 2c were supported.

Furthermore, dependence (B = −0.372, *p* < 0.001), emotional energy (B = −0.153, *p* < 0.05), and relatedness (B = −0.216, *p* < 0.001) reduced employee cyberloafing at work, respectively. AI identity’s three dimensions—dependence, emotional energy, and relatedness—collectively reduce cyberloafing by reinforcing work-focused behaviors (Carter & Grover, 2015; Kopp et al., 2021). Dependence decreases non-work internet use as employees prioritize AI-assisted tasks, while emotional energy enhances work engagement through confidence and trust (Carter et al., 2020). Relatedness fosters psychological bonding with AI systems, making employees more accountable and less likely to engage in cyberloafing (Kopp et al., 2021). Together, these dimensions strengthen professional identity alignment, naturally reducing counterproductive online activities during work hours. Hypothesis 3a, 3b, and 3c were confirmed. In the absence of the mediator, the relationship between human–AI collaboration and cyberloafing was significant (B = −0.279, *p* < 0.001). However, upon incorporating the mediating factor of AI identity, the relationship between these two variables became non-significant (B = −0.079, *p* = 0.249), thereby suggested that AI identity fulfilled a complete mediating role.

**Figure 2:**
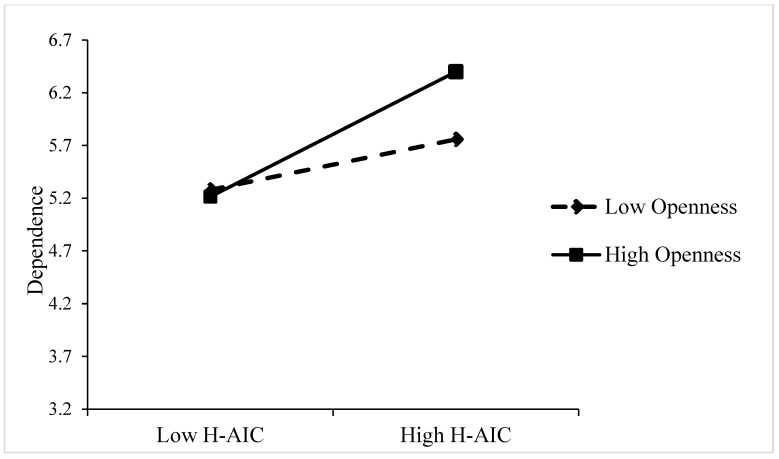
The moderating effect of openness between human–AI collaboration (E-AIC) and dependence.

**Figure 3:**
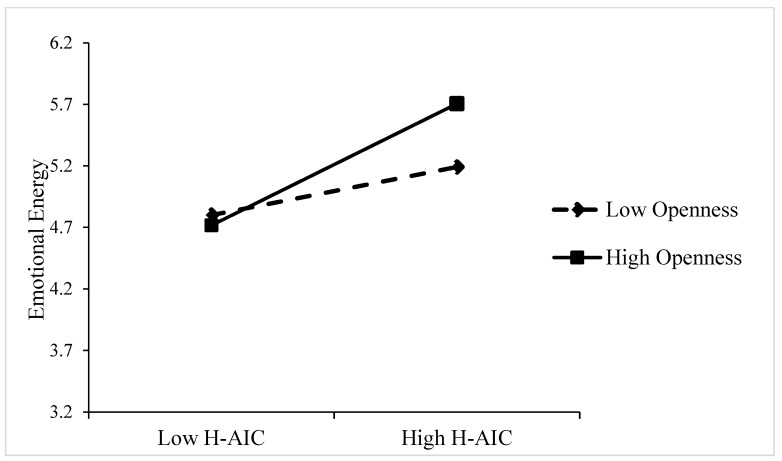
The moderating effect of openness between human–AI collaboration (E-AIC) and emotional energy.

**Figure 4:**
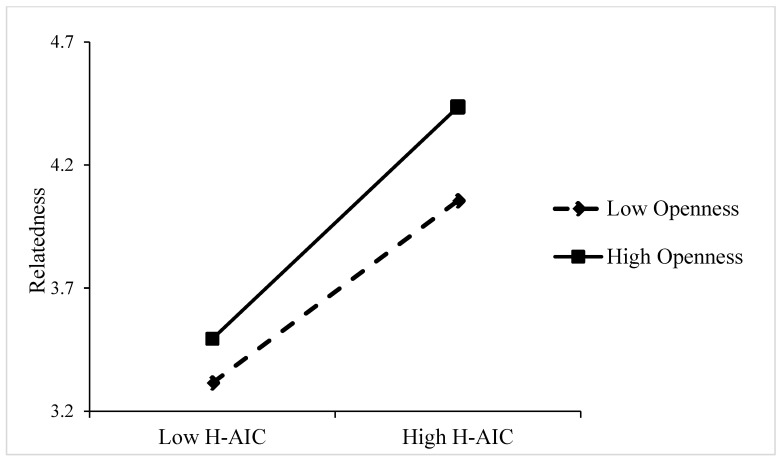
The moderating effect of openness between human–AI collaboration (E-AIC) and relatedness.

*Moderation analyses.* As illustrated in Table 3, the data demonstrated a substantial moderating effect of openness. The interaction term between human–AI collaboration and openness had significant effects on dependence (B = 0.352, *p* < 0.001), emotional energy (B = 0.299, *p* < 0.001), and relatedness (B = 0.101, *p* < 0.05). Openness moderates the relationship between human–AI collaboration and AI identity by amplifying employees’ receptivity to AI (Barford & Smillie, 2016; Tang et al., 2022). To facilitate a more intuitive analysis of the interaction, a simple slope was plotted for the interaction effect, showing low (–1 SD) and high (+1 SD) levels of openness. In comparison to employees exhibiting low levels of openness, those demonstrating high levels of openness were more likely to engender a greater degree of dependence, emotional energy, and relatedness in AI collaboration work (see Figure 2, Figure 3 and Figure 4). Highly open employees demonstrate greater cognitive adaptability and technological curiosity, enabling them to more readily develop dependence on AI’s capabilities (Budhwar et al., 2022; Zhang et al., 2025). They can experience stronger emotional energy from AI interactions and establish deeper relatedness through active exploration of AI systems (Nusbaum & Silvia, 2011; Ma et al., 2024). Consequently, openness intensifies the positive impact of human–AI collaboration on all three AI identity dimensions. Hypothesis 4a, 4b, and 4c were supported. To further explore how openness affects cyberloafing by influencing AI identity, we conducted an indirect-effect analysis (see Table 4). At a low level of openness (M − 1SD), the effects of human–AI collaboration on cyberloafing through dependence (95% CI [−0.032, 0.111], including 0) and emotional energy (95% CI [−0.009, 0.042], including 0) were not significant. As the level of openness increased (M +1SD), the effect of human–AI collaboration on cyberloafing through dependence (95% CI [−0.313, −0.122], not including 0) and emotional energy (95% CI [−0.134, −0.002], not including 0) became significant. Meanwhile, relatedness was significant at both high (95% CI [−0.126, −0.018], not including 0) and low (95% CI [−0.171, −0.044], not including 0) levels of openness. Therefore, the level of openness will affect the outcome of the mediating effect. Hypothesis 5a, 5b, and 5c were confirmed.

## 4. Discussion

Previous research has scarcely explored cyberloafing in AI collaborative work environments, leaving open the question of whether employees collaborating with AI engage in more cyberloafing. Digital transformation has increased employees’ internet access opportunities, creating the potential for cyberloafing (Lai et al., 2025; Tandon et al., 2022). Collaboration with AI has made cyberloafing a focal point of concern for organizations and managers (Zhang et al., 2025). Drawing upon SIT, we explored three dimensions of AI identity and its mediating role between human–AI collaboration and cyberloafing. We have validated the proposed hypotheses through a three-wave lagged study. The findings demonstrated that human–AI collaboration exerted a significant effect on employee cyberloafing within the workplace, particularly by enhancing their AI identity (including dependence, emotional energy, and relatedness). Specifically, the nature of workplace collaboration has fundamentally shifted from traditional human-to-human partnerships to human–AI teamwork (Chowdhury et al., 2022; Seeber et al., 2020). This creates distinct social categorization of AI collaborators and traditional workers under SIT. AI collaboration fosters positive work experiences and enhances group identity through superior performance outcomes, fulfilling social identity and social comparison. Therefore, AI collaboration helps enhance individuals’ AI identity, allowing them to focus on matters within their job role rather than spending time and energy on cyberloafing. Furthermore, we delved into the moderating role of openness, which amplified the positive effect of human–AI collaboration. Employees who exhibit high openness are more likely to accept AI and perceive it as an integral member of the work team (Chakraborty et al., 2023; Slobodskaya & Kornienko, 2021). They seamlessly integrate AI into their personal work identity, perceiving it as a pivotal element for team success. This heightened identification and sense of belonging with AI, in turn, serves to mitigate employees’ tendency to engage in cyberloafing during work hours.

### 4.1. Theoretical Implications

First, this research explores the effect of AI collaboration on employee cyberloafing, supplements relevant research, and extends SIT to the field of AI. AI has had a considerable effect on traditional work modes. A salient transformation is the evolution of the nature of collaboration. Conventional work is characterized by human-to-human partnerships, while AI collaboration is increasingly incorporating AI as a partner or team member (Chowdhury et al., 2022; Seeber et al., 2020). This provides a basis for group distinction between AI collaborators and traditional workers. AI collaboration can inspire employees’ positive views and work experiences of AI (Chowdhury et al., 2022; Sowa et al., 2021). This helps to enhance their sense of identity as members of the AI collaboration group. The efficiency and results of AI collaborators are better than those of traditional workers, which leads to social comparisons among them. Individuals who collaborate with AI fit SIT’s definitions of social categorization, social identity, and social comparison. At the same time, cyberloafing has historically been a focal point for regulatory oversight by organizations and managers. However, there has been a paucity of research examining the phenomenon of cyberloafing in the context of AI collaboration (Glassman et al., 2015; Koay et al., 2022). Consequently, this research broadens the application scope of SIT and establishes a new reference for future research on the relationship between AI collaboration and cyberloafing.

Second, this research explores the relationship between AI collaboration and cyberloafing from the perspective of AI identity, emphasizing the important role of AI identity in AI collaboration. Previous research has mostly focused on traditional interpersonal interactions or group identity (Brown, 2000; Hornsey, 2008). This research extends the concept of IT identity to the domain of AI collaboration, revealing how employees form a new identity through collaboration with AI. We contribute to the extant literature by offering a more nuanced understanding of AI identity. In addition, we have separately explored the three dimensions of AI identity—dependence, emotional energy, and relatedness, and their effects on cyberloafing. By elucidating the mediating role of AI identity, this research provides substantial support for understanding how employees establish connections with AI and form an identity in their work. Furthermore, this research offers novel directions for future research.

Third, we emphasize the pivotal role of openness in the AI collaborative work mode. Openness, as an integral component of personality, exerts a substantial moderating effect on employees’ collaboration with AI. This finding underscores the significance of openness in human–AI collaboration and provides a pivotal focal point for comprehending employees’ acceptance of and adaptation to AI collaboration. This accentuates the necessity for organizations and managers to prioritize the “human” factor in the human–AI collaboration process. It is imperative to recognize that in order to optimize the beneficial role of AI collaboration, it is essential to not only prioritize the advancement of AI development or human capabilities, but also consider both individual traits and the situation of AI collaboration. Through the strategic selection of employees who possess personality traits that are conducive to AI collaboration, such as those who exhibit high openness, it is possible to foster enhanced interaction and collaboration between humans and AI.

### 4.2. Practical Implications

First, we explore the effect of AI collaboration on cyberloafing and offer valuable practical insights for organizations seeking to reduce cyberloafing and promote effective AI collaboration. Effective AI collaboration has the potential to enhance work efficiency and task execution quality, while also reducing unnecessary employee cyberloafing, thereby contributing to organizational discipline and optimizing the work environment. To this end, organizations must proactively adopt an AI collaborative work model, integrating it into employees’ daily operations through meticulous scientific planning and implementation. It is imperative for managers to recognize the potential of AI in guiding employee behavior. By equipping employees with the necessary skills through training, managers can foster effective collaboration with AI, thereby mitigating the potential for cyberloafing stemming from maladjustment or resistance.

Second, organizations and managers must implement measures to cultivate employees’ AI identity, thereby reducing cyberloafing. Organizations can cultivate employees’ comprehension of AI’s role in collaboration through meticulously designed training and promotional initiatives. This can foster a profound realization that AI is not merely a technical apparatus, but a potent collaborator in enhancing work efficiency and quality (Jia et al., 2023; Kong et al., 2023). In such cases, AI emerges as the preferred assistant, mitigating the occurrence of internet browsing behavior stemming from tedious tasks or mounting stress. Concurrently, organizations must prioritize the emotional well-being of their employees, endeavoring to nurture a profound emotional connection with AI. Organizations can build a supportive and inclusive work environment to help employees mitigate the negative effects of emotional alienation or work fatigue. Furthermore, managers can enhance the relatedness between employees and AI by optimizing the interaction design of AI to ensure its enhanced user-friendliness, intelligence, and ease of use. This will reduce cyberloafing caused by emotional disconnection or work fatigue.

Lastly, organizations and managers should prioritize cultivating and selecting employees with openness, as this trait has been shown to significantly moderate the positive effect of AI collaboration. Employees who exhibit high openness levels have a higher acceptance of new technologies and are more willing to try to explore the potential of AI (Barford & Smillie, 2016; Tang et al., 2022). This can better leverage the advantages of AI collaboration. Managers should allocate resources to the cultivation and selection of employees with openness during recruitment and training processes. At the same time, organizations and managers can enhance employees’ tolerance and acceptance of AI collaboration through training, helping them quickly adapt to the working mode of AI collaboration. Moreover, the implementation of incentive mechanisms by managers can encourage employee participation in AI-related training and practice, thereby facilitating efficient AI collaboration (Tang et al., 2022). This, in turn, enables employees to swiftly fulfill their task requirements by leveraging the capabilities of AI, thereby reducing cyberloafing by avoiding the inefficient use of time and energy on information searches and filtering.

### 4.3. Limitations and Future Directions

First, the samples in this study come from an online platform, and all participants are employees in Chinese organizations. The source and scope of the sample are limited, ignoring the possible effect of factors such as the regional culture and national technological development level (Duan et al., 2025). Future research should expand the sample range and collect cross-cultural sample data from both developed and developing countries to improve the generalizability and reliability of the results (Wu et al., 2024a).

Second, it must be acknowledged that the data in this research were predominantly collected through self-reports, which limits its ability to accurately reflect causal relationships between variables. This research only entailed a multi-wave study. Future research can use a combination of multi-wave and scenario experiments to further verify the causal relationship between variables. Furthermore, in addition to considering employees’ evaluation of their own behavior, their behavior can also be evaluated by others (e.g., leaders or colleagues).

Finally, while we have examined the effect of AI collaboration on cyberloafing from the perspective of AI identity, it is important to note that the mechanisms and outcomes of AI collaboration are multifaceted. In this vein, future research endeavors may benefit from incorporating additional variables to facilitate more profound explorations, such as those related to emotions, attitudes, and cognitive processes (Wu et al., 2022).

## 5. Conclusions

As more and more people engage in collaborative endeavors with AI in their professional pursuits, it becomes imperative to examine the emergence of AI identity among these individuals and its subsequent impact on their behavior. Drawing from SIT, our research offers a novel perspective on the exploration of human–AI collaboration. Specifically, we propose an examination of AI identity as a pivotal factor in this relationship. Through a three-wave study, we have obtained a more profound understanding of the effect of AI collaboration on employees’ AI identity. And AI identity exerts a subsequent influence on cyberloafing. Our findings suggest that the relationship between human–AI collaboration and cyberloafing is moderated by openness. Employees who exhibit higher levels of openness are more likely to collaborate with AI and benefit from AI collaboration, thereby significantly increasing AI identity and reducing cyberloafing. This research assists organizations and managers in comprehending the effect of human–AI collaboration from an AI identity perspective, and in implementing measures to promote collaboration between employees and AI. This will reduce cyberloafing and enable the attainment of the dual objective of benefiting both the organization and its employees.

## Figures and Tables

**Table 1:** Means, SDs, and correlation analysis.

Variables	M	SD	1	2	3	4	5	6	7	8	9	10
1 Gender	1.504	0.501	1									
2 Age	33.780	9.401	0.023	1								
3 Education	4.428	1.376	0.332 **	−0.030	1							
4 Tenure (of years)	10.135	6.651	−0.030	0.418 **	−0.239 **	1						
5 H-AIC	3.815	0.857	0.219 **	−0.128 *	0.493 **	−0.208 **	1					
6 Dependence	3.310	1.320	−0.115 *	−0.316 **	0.055	−0.353 **	0.176 **	1				
7 Emotional Energy	3.617	0.956	−0.090	−0.283 **	0.052	−0.284 **	0.179 **	0.709 **	1			
8 Relatedness	3.885	0.906	0.194 **	−0.110 *	0.421 **	−0.185 **	0.509 **	0.248 **	0.216 **	1		
9 Openness	3.892	0.945	0.271 **	−0.042	0.576 **	−0.181 **	0.427 **	0.162 **	0.161 **	0.478 **	1	
10 Cyberloafing	2.642	1.069	0.071	0.195 **	−0.052	0.249 **	−0.216 **	−0.614 **	−0.513 **	−0.320 **	−0.131 *	1
AVE							0.662	0.862	0.826	0.786	0.742	0.700
CR							0.907	0.949	0.935	0.917	0.920	0.962

Note: N = 381; * *p* < 0.05; ** *p* < 0.01. H-AIC = human–AI collaboration.

**Table 2:** Results of mediation analysis.

Variables	Dependence		Emotional Energy		Relatedness		Cyberloafing			
	*B*	*SE*	*B*	*SE*	*B*	*SE*	*B*	*SE*	*B*	*SE*
Control Variable										
Gender	−0.362 **	0.126	−0.217 *	0.096	0.074	0.076	0.213	0.110	0.061	0.087
Age	−0.026 **	0.008	−0.019 **	0.006	−0.004	0.006	0.010	0.006	−0.003	0.005
Education	−0.038	0.062	−0.028	0.048	0.137 **	0.042	0.056	0.046	0.068	0.040
Tenure (of years)	−0.051 ***	0.012	−0.027 **	0.009	−0.005	0.008	0.030 ***	0.009	0.006	0.008
Independent Variable										
H-AIC	0.228 **	0.082	0.180 **	0.064	0.406 ***	0.063	−0.279 ***	0.071	−0.079	0.069
Mediator										
Dependence									−0.372 ***	0.047
Emotional Energy									−0.153 *	0.067
Relatedness									−0.216 ***	0.054
*R* ^2^	0.190		0.142		0.302		0.115		0.425	
*F*	17.595 ***		12.401 ***		32.488 ***		9.781		34.304 ***	

Note: N = 381; * *p* < 0.05; ** *p* < 0.01; *** *p* < 0.001. H-AIC = human–AI collaboration.

**Table 3:** Results of moderated mediation analysis.

Variables	Dependence		Emotional Energy		Relatedness		Cyberloafing	
	*B*	*SE*	*B*	*SE*	*B*	*SE*	*B*	*SE*
Control Variable								
Gender	−0.342 **	0.127	−0.196 *	0.094	0.050	0.081	0.037	0.090
Age	−0.028 ***	0.007	−0.020 ***	0.005	–0.005	0.004	−0.002	0.005
Education	−0.083	0.058	−0.057	0.043	0.063	0.037	0.042	0.041
Tenure (of years)	−0.048 ***	0.010	−0.025 **	0.008	–0.003	0.007	0.006	0.007
Independent Variable								
H-AIC	0.240 **	0.083	0.196 **	0.061	0.370 ***	0.053	−0.124 *	0.062
Mediator								
Dependence							−0.362 ***	0.047
Emotional Energy							−0.125 *	0.063
Relatedness							−0.216 ***	0.058
Moderator Variable								
Openness	0.288 ***	0.080	0.216 ***	0.059	0.280 ***	0.051	0.012	0.059
H-AIC × Openness	0.352 ***	0.076	0.299 ***	0.057	0.101 *	0.049	−0.180 **	0.056
*R* ^2^	0.250		0.218		0.358		0.442	
*F*	17.759 ***		14.835 ***		29.648 ***		29.249 ***	

Note: N = 381; * *p* < 0.05; ** *p* < 0.01; *** *p* < 0.001. H-AIC = human–AI collaboration.

**Table 4:** Conditional indirect effects of human–AI collaboration (H-AIC) on cyberloafing via dependence, emotional energy, and relatedness.

Pathway	Openness	Effect	BootSE	Boot 95% CI
Direct Effect				
H-AIC → Cyberloafing	M − 1SD	0.046	0.073	[−0.098, 0.190]
	M + 1SD	−0.295	0.090	[−0.471, −0.119]
Indirect Effect				
H-AIC → Dependence → Cyberloafing	M − 1SD	0.034	0.036	[−0.032, 0.111]
	M + 1SD	−0.207	0.049	[−0.313, −0.122]
H-AIC→ Emotional Energy → Cyberloafing	M − 1SD	0.011	0.013	[−0.009, 0.042]
	M + 1SD	−0.060	0.034	[−0.134, −0.002]
H-AIC→ Relatedness → Cyberloafing	M − 1SD	−0.059	0.028	[−0.126, −0.018]
	M + 1SD	−0.100	0.033	[−0.171, −0.044]

## Data Availability

The data are not publicly available due to privacy or ethical restrictions. The data presented in this study are available on request from the corresponding author.
